# NTRU-Like Random Congruential Public-Key Cryptosystem for Wireless Sensor Networks †

**DOI:** 10.3390/s20164632

**Published:** 2020-08-17

**Authors:** Anas Ibrahim, Alexander Chefranov, Nagham Hamad, Yousef-Awwad Daraghmi, Ahmad Al-Khasawneh, Joel J. P. C. Rodrigues

**Affiliations:** 1 Department of Computer Systems Engineering, Palestine Technical University—Kadoorie, Tulkarm 7, Palestine; y.awwad@ptuk.edu.ps; 2 Department of Computer Engineering, Eastern Mediterranean University, Famagusta 99628, North Cyprus via Mersin 10, Turkey; alexander.chefranov@emu.edu.tr; 3 Department of Information Technology, Palestine Technical University—Kadoorie, Tulkarm 7, Palestine; nagham.hamad@ptuk.edu.ps; 4 Irbid National University, P.O. Box 2600, Irbid 21110, Jordan; akhasawneh@hu.edu.jo; 5 Department of Computer Information System, Hashemite University, P.O. Box 150459, Zarqa 13115, Jordan; 6 Federal University of Piauí, Teresina 64049-550, PI, Brazil; joeljr@ieee.org; 7 Instituto de Telecomunicações, 6201-001 Covilhã, Portugal

**Keywords:** wireless sensor network, random congruential public-key cryptosystem, lattice, NTRU, polynomial, lattice basis reduction attack, LLL algorithm, Gaussian lattice reduction, IND-CCA2 security

## Abstract

Wireless sensor networks (WSNs) are the core of the Internet of Things and require cryptographic protection. Cryptographic methods for WSN should be fast and consume low power as these networks rely on battery-powered devices and microcontrollers. NTRU, the fastest and secure public key cryptosystem, uses high degree, *N*, polynomials and is susceptible to the lattice basis reduction attack (LBRA). Congruential public key cryptosystem (CPKC), proposed by the NTRU authors, works on integers modulo *q* and is easily attackable by LBRA since it uses small numbers for the sake of the correct decryption. Herein, RCPKC, a random congruential public key cryptosystem working on degree N=0 polynomials modulo *q*, is proposed, such that the norm of a two-dimensional vector formed by its private key is greater than $\sqrt{q}$. RCPKC works as NTRU, and it is a secure version of insecure CPKC. RCPKC specifies a range from which the random numbers shall be selected, and it provides correct decryption for valid users and incorrect decryption for an attacker using LBRA by Gaussian lattice reduction. RCPKC asymmetric encryption padding (RAEP), similar to its NTRU analog, NAEP, is IND-CCA2 secure. Due to the use of big numbers instead of high degree polynomials, RCPKC is about 27 times faster in encryption and decryption than NTRU. Furthermore, RCPKC is more than three times faster than the most effective known NTRU variant, BQTRU. Compared to NTRU, RCPKC reduces energy consumption at least thirty times, which allows increasing the life-time of unattended WSNs more than thirty times.

## 1. Introduction

Wireless sensor networks (WSNs) play an important role in the development of the Internet of Things (IoT). WSNs consist of a large number of sensor nodes, battery-supplied devices with a limited memory and computational power microcontroller. WSNs are used widely, e.g., in environmental practices, health, industrial control, military [1], multimedia networks [2], and smart grid networks [3]. WSNs need security and confidentiality since sensitive information is stored, processed, or transferred by sensor nodes [4]. Therefore, cryptographic schemes efficiently working on limited WSN microcontrollers are demanded [5]. Furthermore, energy savings is very important for WSNs [6]. NTRU [7,8] is a public key cryptosystem (PKC) standardized as IEEE P1363.1 and is faster than RSA and ECC [9], and it is applicable to WSNs [10]. Contrary to RSA and ECC working with big numbers and homomorphic only in one operation, multiplication and addition, respectively, NTRU works with high degree, *N*, polynomial rings and is homomorphic with respect to both multiplication and addition [11]. These features of NTRU allow its use in various applications, such as authentication for smart cards [12], encryption of user data in smart monitoring systems [13], securing of SMS [14], mutual authentication and key agreement for wireless communications [15], embedded systems including microcontrollers and FPGAs [16], Internet of Things devices [17], and NTRU hardware implementation [18]. The NTRU model expects that the public key is used for encryption only by a public user (sender), whereas the private key is used for decryption by the key’s owner (receiver).

NTRU and its known variants [7,8,19,20,21,22,23,24,25,26,27,28,29,30,31,32,33,34,35,36,37,38], shown in Section 2, work with degree *N* polynomials. The main problem NTRU faces is that it is susceptible to the lattice basis reduction attack (LBRA) using the Gaussian lattice reduction (GLR) algorithm for two-dimensional lattices and the LLL algorithm for higher dimensions [39]. The LBRA using LLL algorithm solves the shortest vector problem (SVP) with exponential in *N* running time revealing the secret key because the private keys are selected as polynomials with small coefficients for the decryption correctness [40]. To overcome the problem of susceptibility, NTRU uses large *N* resulting in high computational complexity [11,41]. Therefore, NTRU variants, shown in Section 2, try minimizing NTRU computational complexity by extending the coefficients of the polynomials used or using matrices of polynomials that allow preserving the security level while decreasing the polynomial degree. The extreme case is a polynomial of zero degree, that is integers modulo q>>1, as used in the congruential public key cryptosystem (CPKC), but CPKC with the NTRU encryption/encryption mechanism is insecure against LBRA by GLR (crackable in about 10 iterations) ([26], pp. 373–376, 451). Therefore, the CPKC is considered as a toy model of NTRU because “it provides the lowest dimensional introduction to the NTRU public key cryptosystem” [26] (p. 374). The insecurity of CPKC stems from the choice of the private keys used as small numbers to provide decryption correctness. If CPKC could be made resistant to GLR attack, it would be the best possible choice for the NTRU modifications. Therefore, we propose a CPKC modification, random CPKC (RCPKC) [42] (we call it here RCPKC.1).

In this paper, an enhanced RCPKC is proposed by specifying a range from which the random numbers shall be selected based on short vectors returned by GLR attack on it. It provides correct decryption for valid users and incorrect decryption for an attacker using GLR. GLR cannot find its private key because it solves SVP returning the shortest in a lattice vector, whereas our private key is in the safe region (above Minkowski’s boundary (27)–(30) for the shortest vector norm of a lattice). On the other hand, the short vectors returned by GLR cannot be used for correct decryption due to our choice of the random numbers. RCPKC is a cryptosystem more secure than NTRU because LBRA is currently considered as one of the most effective attacks against NTRU, and also, a number of other attacks on NTRU are not applicable to RCPKC, whereas RCPKC’s resistance to other known attacks on NTRU is similar to that of NTRU. RCPKC is about 27 times faster in encryption and decryption than NTRU. Simplicity, speed, and security make RCPKC a good candidate cryptosystem for WSNs. The paper’s contribution can be summarized as follows:

- RCPKC, an NTRU-like cipher variant resistant to lattice based attacks, is proposed with enhanced security compared to RCPKC.1 [42].
- The hardness of the RCPKC one-way (OW) function is proven.
- RCPKC symmetric encryption padding (RAEP) is IND-CCA2 secure is proven under the assumption of the hardness of inverting an associated one-way function.
- RCPKC’s performance is justified through implementation and comparison with the state-of-the-art ciphers.
- RCPKC’s better applicability than NTRU to WSNs is proven.

The rest of the paper is organized as follows. In Section 2, known NTRU variants are presented. In Section 3, an overview of NTRU, NTRU AEP (NAEP), the IND-CCA2 security of NAEP, and CPKC is given, and formulas for CPU power consumption calculation are introduced. LBRA by GLR on CPKC is described, and Minkowski’s second theorem is presented in Section 4, used to define a region where GLR attack against the CPKC private key/message fails. In Section 5, RCPKC is presented. In Section 6, the RCPKC security comparison versus NTRU is conducted. In Section 7, the RCPKC OW function and RCPKC asymmetric encryption padding (RAEP) IND-CCA2 security are considered. In Section 8, the RCPKC performance comparison versus NTRU and its variants is presented, and the RCPKC versus NTRU power consumption is studied. Section 9 concludes the paper.

## 2. Review of Known NTRU Variants

Many variants of NTRU have been proposed and studied recently, targeting further decreasing its computational complexity. All these variants work with polynomials and mainly differ in the choice of their coefficients, the ring defining polynomial, or the polynomials used as the entries of such structures as matrices. The NTRU variants’ overview follows.

**NTRU variants differing in the choice of their coefficients.** In [27], an NTRU variant, ETRU, was proposed working with polynomials over Eisenstein integer coefficients and was faster than NTRU in encryption/decryption by 1.45/1.72 times. Karbasi and Atani [28] modified ETRU, called ILTRU [28], so that it works with irreducible cyclotomic polynomial over Eisenstein integer coefficients. An NTRU variant, BITRU, working with polynomials over so-called binary numbers, usually known as complex numbers, was proposed in [20]. An NTRU variant, QTRU, working with polynomials over hyper-complex four-component numbers, quaternions, was proposed in [30]. Furthermore, Bagheri and colleagues proposed an NTRU variant, BQTRU, working over quaternions, but with bivariate polynomials, seven times faster than NTRU in encryption [21]. A variant of NTRU working with polynomials over eight-component hyper-complex numbers, octonions, called OTRU, was proposed in [29]. In [34], an NTRU variant, HXDTRU, was proposed working with polynomials over 16-component hyper-complex numbers, hexadecnions, also known as sedenions [19]. Furthermore, a variant of NTRU working with polynomials over 16-component hyper-complex numbers, sedenions, was proposed in [31]. A variant of NTRU, called CTRU, working with polynomials, the coefficients of which are also polynomials in one variable over a binary field, was proposed in [24]. Furthermore, a variant of NTRU working with polynomials, the coefficients of which are polynomials in one variable over a rational field, called BTRU, was proposed in [32].

**NTRU variants working with different rings.** An NTRU variant that works with polynomials with prime cyclotomic rings was proposed in [35]. A variant of NTRU working with non-invertible polynomials was proposed in [22].

**NTRU variants working with polynomials inside more complicated structures.** An NTRU variant working with square matrices of polynomials was proposed in [23] and was shown to be 2.5 times better than NTRU encryption and decryption time. An NTRU variant, called NNRU, working with polynomials also being entries of square matrices forming a non-commutative ring, was proposed in [33]. Apart from the polynomial variants, an NTRU-like cipher over the ring of integers, called ITRU, was proposed in [25] without referencing CPKC [26]. In ITRU [25], Table 1, p. 34, the NTRU model specified above was given, but a model for the proposed ITRU was not defined. Its Algorithm 1, [25], p. 35, describes the key generation, and hence, it shall be made by the key owner (receiver). On the other hand, in Section 4. A, Parameter selection, [25], p. 37, the most important parameter, *q*, was selected by the sender (which encrypts a message using the public key, $h^{\prime }=$ 423,642, and random value, $r^{\prime }=19$, in [25], (19), p. 37 with the help of the private keys, $f^{\prime },g^{\prime }$, which contradicts the NTRU model: the secret key is known to only the key owner that uses the private key only for decryption, whereas the public key is used for encryption by the public user only.

Thus, the NTRU variants try minimizing NTRU’s computational complexity by extending coefficients of the polynomials used or using matrices of polynomials that allow preserving the security level while decreasing the polynomial degree because operations with high-degree polynomials are time-consuming. However, these variants are still susceptible to LBRA using LLL, but with less complexity than NTRU has. Furthermore, ITRU can be used by the key owner only for encryption and decryption messages, but cannot be used by a public user knowing the public key only; hence, it is not compatible with the NTRU model of use.

## 3. Preliminaries

### 3.1. Overview of NTRU

NTRU [7,8] uses the rings:

$$R_{q}=\frac{Z_{q}\left[x\right]}{x^{N}-1},R_{p}=\frac{Z_{p}\left[x\right]}{x^{N}-1},$$

elements of which are polynomials modulo $x^{N}-1$ with coefficients in $Z_{q},Z_{p}$, respectively, where p=2, q>4d+1 is prime, N>3k+8 is prime, *k* is the security parameter, and *d* is the minimal integer such that N/2d/2/sqrt(N)>2k, where mn is the number of combinations of *n* elements out of *m*.

Secret polynomials, *f* and *g*, are binary polynomials from $D_{f}$ and $D_{g}$, with $d_{f}=d_{g}=d$ coefficients equal to one. Both *f* and *g* are invertible modulo *q*. Public key, *h*, is defined as:

(1) $$h=p\cdot F_{q}\cdot g\;\mathrm{mod}\;q,$$

where $F_{q}$ is the inverse of *f* modulo *q*. A binary message, $m=D_{m}\in R_{p}$, is encrypted using a random binary polynomial *r* from $D_{r}$ with the $d_{r}=\left\lfloor N/2\right\rfloor$ ones as follows:

(2) $$e=F_{h,p}^{NTRU}\left(m,r\right)=p\cdot r\cdot h+m\;\mathrm{mod}\;q.$$

NTRU decryption consists of two steps:

**Step 1**: The private key, *f*, is applied to (2):

(3) $$\begin{array}{ccc} a & = & f\cdot e\;\mathrm{mod}\;q\\ & = & p\cdot r\cdot g+f\cdot m. \end{array}$$

**Step 2**: The inverse of *f* modulo *p* is applied to (3) after the polynomial *a* is centered using the center() function. An implementation of center(), called center1(), provided in [43] (p. 4), follows:

1. Calculate m(1) as e(1)−p·r(1)·h(1), reduced to the interval,
  (4) $$\frac{N-q}{2}\leq m\left(1\right)<\frac{N+q}{2}.$$
2. Denote *a* reduced to the interval [0,q−1] by $\underline{a}$. The underline is intended to indicate the minimal possible interval.
3. Calculate a(1). This will differ from p·r(1)·h(1)+f(1)·m(1) by k·q, for some integer, *k*.
4. Add *q* to the lowest *k* entries of *a* to obtain *a* reduced into the correct interval.

NTRU decryption fails if the following condition does not hold,

(5) Width(p·g·r+f·m)<q.

where $Width\left(p\left(x\right)\right)=\max_{i=0,\cdots ,N-1}\left(p_{i}\right)-\min_{i=0,\cdots ,N-1}\left(p_{i}\right)$. Application of the center() function to the left-hand side (LHS) of (3) makes the second equality in (3) true under Condition (5). For the conditions imposed on NTRU parameters described above, in particular, p=2,q>4d+1, the second equality in (3) holds, and there is no need for centering.

### 3.2. NTRU Asymmetric Encryption Padding IND-CCA2 Security

NTRU asymmetric encryption padding (NAEP) has been proven IND-CCA2 secure [7]. In Section 3.2.1, NAEP is introduced, and in Section 3.2.2, its IND-CCA2 security is discussed.

#### 3.2.1. NAEP Description

NAEP uses a function,

(6) compress(p(x))=p(x)modqmod2,

where p(x) is a polynomial. NAEP encryption is introduced in Algorithm 1.
**Algorithm 1:** NAEP encryption.
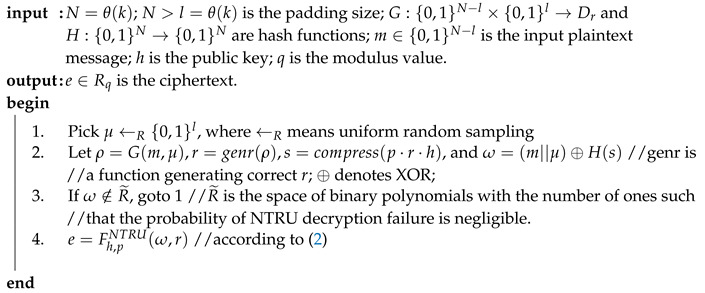


The compress() binary string result is used in Step 2 of NAEP encryption to hide the padded message by the XOR operation and hashing. NAEP decryption is introduced in Algorithm 2.
**Algorithm 2:** NAEP decryption.
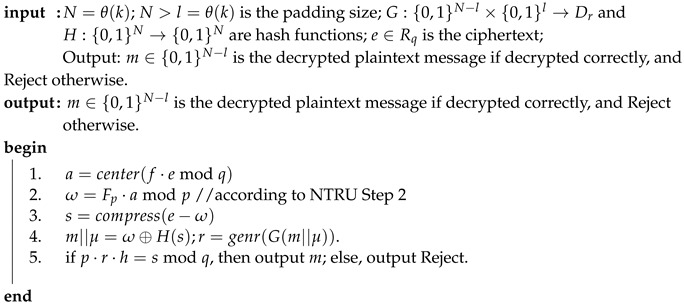


#### 3.2.2. NAEP IND-CCA2 Security

NAEP has been proven to be IND-CCA2 secure [7].

**Definition** **1.** *[7] (p. 3): A time τ algorithm A is a (τ;ϵ)-chosen ciphertext algorithm, with advantage ϵ in attacking a randomized encryption scheme (K,E,D) if there is a pair of sub-algorithms*

$$A_{1}:PK\to M\times M\times S,$$

$$A_{2}:C\times S\to \left\{0,1\right\},$$

*such that if $\left(m_{0},m_{1},s\right)\leftarrow A_{1}\left(pk\right)$, then:*

$$Prob\left(A_{2}\left(c^{*},s\right)=b\right)-\frac{1}{2}=\frac{1}{2}\epsilon ,$$

where $c^{*}\leftarrow E\left(m^{*},r^{*}\right),$ for some $r^{*}\in R_{E}$ and $m^{*}=m_{b^{*}}$ for some $b^{*}\in \left\{0,1\right\}$. This probability is defined over the choice of $r\leftarrow_{R}R_{E},b^{*}\in \left\{0,1\right\}$, and $k\in R_{K}$, where $R_{E}$ and $R_{K}$ are defined below.

The algorithms $\left(A_{1},A_{2}\right)$ have access to a decryption oracle D, which they can call on all but the challenge ciphertext $c^{*}$, but they must make all hash function calls to $H_{1},\ldots ,H_{n}$ public.

An encryption scheme is IND-CCA2 secure if there exist no polynomial (on security parameter) time adversary with a non-negligible advantage. Key generation, encryption, and decryption algorithms are formalized as follows [7]. For a given parameter set *P*, the encryption scheme is specified by three algorithms:

$$K:R_{K}\to PK\times SK,$$

$$E:PK\times M\times R_{E}\to C,$$

D:SK×C→M,

called the key generation, encryption, and decryption algorithms, respectively. The spaces $R_{K},PK,SK,M,R_{E},C$ are called the key-gen randomness, public key, secret key, message, encryption randomness, and ciphertext space, respectively.

If $\left(pk_{k},sk_{k}\right)\leftarrow K\left(k\right)$, then the algorithms should satisfy:

$$D(sk_{k},E\left(pk_{k},m,r\right))=m$$

for all $k\in R_{K},m\in M$ and $r\in R_{E}$.

NTRU key, encryption, and decryption procedures and respective spaces are defined in Section 3.1 according to [7]. Polynomials used in NAEP [7,8] for keys are invertible. The NTRU one-way problem is defined as follows:

**Definition** **2.** *NTRU-OW problem: For a parameter set, $P_{NTRU}$, we denote by $Succ_{NTRU}^{OW}\left(A,P_{NTRU}\right)$ the success probability of a probabilistic polynomial time (PPT) adversary, A, for finding a pre-image of $F_{h,p}^{NTRU}$,*

$$Succ_{NTRU}^{OW}\left(A,P_{NTRU}\right)=\mathrm{Pr}\left(\begin{array}{c} \left(m^{\prime },r^{\prime }\right)\leftarrow A\left(e,h\right)\\ 𝘴𝘶𝘤𝘩\;𝘵𝘩𝘢𝘵\;F_{h}^{NTRU}\left(m^{\prime },r^{\prime }\right)=e \end{array}\right).$$

**Assumption** **1.** *NTRU-OW assumption: For every PPT adversary, A, solving the NTRU-OW problem, there exists a negligible function, $v_{A}\left(k\right)$, such that for sufficiently large k, it holds:*

$$Succ_{NTRU}^{OW}\left(A,P_{NTRU}\right)\leq v_{A}\left(k\right).$$

NTRU variants [7,40] can fail (see Section 6.5); hence, it was assumed in [7] that the failure probability is negligible. Under these assumptions, the IND-CCA2 security of NAEP was proven in [7], Corollary 1.

### 3.3. Overview of CPKC

Two secret integers, *f* and *g*, are defined as follows:

(7) $$f<\sqrt{q/2},\;\sqrt{q/4}<g<\sqrt{q/2},$$

(8) gcd(f,qg)=1,

where *q* is a public integer.

The first secret value, *f*, has inverses modulo *g* and *q*, that is $F_{g}$ and $F_{q}$, respectively, by virtue of (8):

(9) $$1=f\cdot F_{g}\;\mathrm{mod}\;g,\;1=f\cdot F_{q}\;\mathrm{mod}\;q.$$

A public value, *h*, is computed using (7) and (9) as follows:

(10) $$h=F_{q}\cdot g\;\mathrm{mod}\;q.$$

Thus, CPKC has the private (secret) key, $SK=(f,g,q,F_{g},F_{q})$, and the public key, PK=(h,q). The plaintext message, *m*, meets the following condition:

(11) $$0<m<\sqrt{q/4}.$$

A random integer, *r*, is chosen as follows:

(12) $$0<r<\sqrt{q/2}.$$

#### 3.3.1. CPKC Encryption

The ciphertext, *e*, is computed using (10)–(12) as follows:

(13) $$e=F_{h}\left(m,r\right)=r\cdot h+m\;\mathrm{mod}\;q.$$

#### 3.3.2. CPKC Decryption

Decryption is described by Steps 1 and 2 below:

**Step 1:** Multiply the ciphertext (13) by *f*, getting:

a=f·emodq

(14) $$=r\cdot f\cdot F_{q}\cdot g+f\cdot m\;\mathrm{mod}\;q.$$

Note that a=r·g+f·m if (the remainder is allowed to be negative):

(15) |r·g+f·m|<q,

where (9), (10) and (13) are used. The CPKC decryption correctness condition (15) holds under Conditions (7), (11) and (12):

$$|r\cdot g+f\cdot m|<\sqrt{q/2}\sqrt{q/2}+\sqrt{q/2}\sqrt{q/4}<q.$$

Thus, the parameters, *f*, *g*, and *r*, are selected small compared to *q* (see (7), (11) and (12)) to meet the CPKC correctness decryption condition (15) used in Step 2 of the decryption.

**Step 2:** Multiply (14) by $F_{g}$, getting:

(16) $$m=a\cdot F_{g}\;\mathrm{mod}\;g,$$

where (9) is used and the contributor with the factor *g* in (14) vanishes due to (15). Numerical Example S1 of CPKC encryption/decryption is in the Numerical Supplementary Materials.

### 3.4. Formulas for CPU Power Consumption Calculation

Power, *P*, and energy, *E*, are measured in watts (*W*) and joules (*J*) [44], respectively, and calculated as follows:

(17) P=V·I,

(18) E=P·T,

where *V* is the potential difference measured in volts (*V*), *I* is the electric current measured in amperes (A), and *T* is the running time in seconds. There are three contributors to the CPU power consumption: dynamic, short-circuit, and power loss due to transistor leakage currents [45]:

(19) $$P_{cpu}=P_{dyn}+P_{sc}+P_{leak}.$$

Power consumption is mainly defined by the dynamic and leakage components [46]. Leakage power, caused by leakage currents, is present in any active circuit independent of clock rates and is calculated as follows [46]:

(20) $$P_{leak}=V\cdot I_{leak},$$

where *V* is the supply voltage and $I_{leak}$ is the leakage current. Dynamic power consumption depends on circuit activity (i.e., transistor switches, changes of values in registers, etc.) and is defined as follows [45]:

(21) $$P_{dyn}=a\cdot C\cdot V^{2}\cdot f,$$

where a is the switching activity factor, *C* is the capacitance measured in farad (*F*), and *f* is the clock frequency measured in hertz (Hz). Mostly, the activity factor is a = 0.5 [47]. MSP430FR5969, a Texas Instruments microcontroller with capacitance C = 20 pF [48] (Tables 5–12), active supply voltage from 1.8,…,3.6 V [48] (p. 1), clock frequency from 1,…,16 MHz [48] (p. 19), $I_{leak}$ =20 nA [48] (Tables 5–11), is used for RCPKC power consumption evaluation in Section 8.2.

## 4. Analysis of LBRA Attack Against CPKC

In this section, LBRA using GLR against the CPKC private key/message is described. Our implementation of GLR attack is shown (Maple 2016.2 is used throughout the paper). A demonstration by an example of how the CPKC private key can be attacked using GLR is presented. Then, a region defined in terms of Minkowski’s second theorem where GLR attack fails is shown.

### 4.1. Lattice Basis Reduction Attack by GLR on CPKC Private Key/Message

In the following, ||x||, (x·y), ⌊a⌉, and *R*, denote the Euclidean norm [49] of the vector *x*, the dot product of the vectors, *x* and *y*, the rounding of the real number, *a*, and the set of real numbers, respectively.

Let $E\left(V_{1},V_{2}\right)\subset R^{2}$ be a two-dimensional lattice with basis vectors, $V_{1}$ and $V_{2}$:

(22) $$E\left(V_{1},V_{2}\right)=\left\{a_{1}V_{1}+a_{2}V_{2}:a_{1},a_{2}\in Z\right\}.$$

The GLR algorithm [26] (p. 437), shown in Code 1, upon termination returns the shortest vector $v_{1}$ in $E(V_{1},V_{2})$.

**Code** **1.** *GLR algorithm pseudocode finding the shortest vector $v_{1}$ of the lattice $E(V_{1},V_{2})$.*

*  **Input**: basis vectors $V_{1}$, $V_{2}$;*

*  **Output**: the shortest vector $v_{1}$ in $E(V_{1},V_{2})$;*

  $v_{1}=V_{1}$*;* $v_{2}=V_{2}$;

  *Loop*

   *If* $\left|\right|v_{2}\left||<|\right|v_{1}\left|\right|$

     *swap* $v_{1}$ *and* $v_{2}$.

   *Compute $m=\lfloor \left(v_{1}\cdot v_{2}\right)/||v_{1}|{|}^{2}\rceil$*.

   *If *m** = 0

    *return the shortest vector $v_{1}$ of the basis, $\left\{v_{1},v_{2}\right\}$*.

    *Replace $v_{2}$ with $v_{2}$-$mv_{1}$.*

  *End Loop*.

The CPKC private key recovery problem can be formulated as the shortest vector problem (SVP) in the two-dimensional lattice, $E(V_{1},V_{2})$. From (10), it can be noticed that for any pair of integers, *F* and *G*, satisfying:

(23) $$G=Fh\;\mathrm{mod}\;q,\;F=O\left(\sqrt{q}\right),\;G=O\left(\sqrt{q}\right),$$

(F,G) is likely to serve as the first two components, *f* and *g*, of the private key, SK [26] (p. 376). Equation (23) can be written as F·h+q·n=G, where *n* is an integer. Therefore, our task is to find a pair of comparatively small by absolute value integers, (F,G), such that:

(24) $$F\cdot V_{1}+n\cdot V_{2}=\left(F,G\right),$$

where $V_{1}=\left(1,h\right)$ and $V_{2}=\left(0,q\right)$ are basis vectors, at least one of them having the Euclidean norm of order O(q). Similarly, the CPKC message recovery problem can be formulated as the SVP in the two-dimensional lattice, $E(V_{1},V_{2})$, where $V_{1}$ and $V_{2}$ are from (24). It can be also noticed from (13) that for any pair of integers, (RR,EM), satisfying:

(25) $$EM=RR\cdot h\;\mathrm{mod}\;q,\;RR=O\left(\sqrt{q}\right),\;EM=O\left(\sqrt{q}\right),$$

(RR,EM) is likely to serve as the vector (r,e−m) since the encryption Equation (13) can be written as r·h+q·n=e−m, where *n* is an integer. Therefore, our task is to find a pair of comparatively small by absolute value integers, (RR,RM), such that:

(26) $$RR\cdot V_{1}+n\cdot V_{2}=\left(RR,EM\right).$$

Our aim is to find the shortest vector *w* from $E(V_{1},V_{2})$ using GLR that might disclose (r,e−m) if *e* and *r* are of the order of $O(\sqrt{q})$. Comparing (24) and (26), it is noticed that they are the same up to the unknowns’ names used, and hence, finding the shortest vector in $E(V_{1},V_{2})$ may reveal either the private key components, (F,G) = (f,g), or the message related vector, (RR,EM)=(r,e−m).

Code S1 in the Supplementary Materials presents our Maple [50] implementation of LBRA by GLR based on Code 1. Example S2 in the Supplementary Materials provides an example of LBRA attack using GLR against CPKC.

This section concludes that CPKC can be easily attacked using GLR. In order to modify CPKC to become resistant to GLR attack, first, in Section 4.2, a region where GLR attack fails is shown.

### 4.2. Region Resistant to GLR Attack on the CPKC Private Key/Message

LBRA by GLR succeeds in finding the CPKC private key, since it, by using the settings (7), is likely the shortest vector in the lattice. Minkowski’s second theorem [51] (p. 35) sets an upper bound for the norm of the shortest nonzero vector, λ, in a two-dimensional lattice:

(27) $$\lambda \leq \sqrt{\lambda_{2}}\mathrm{Vol}{\left(L\right)}^{1/2},$$

where $\lambda_{2}$=$2/\sqrt{3}\approx$ 1.154 is Hermite’s constant [51] (p. 41) and Vol(L) is the volume of the lattice, which is equal to *q* for the lattice $L=E(V_{1},V_{2})$ where $V_{1}$ and $V_{2}$ are defined in (24). Therefore, (27) can be written as follows:

(28) $$\lambda \leq \alpha \sqrt{q},$$

where α = $\sqrt{\lambda_{2}}\approx$ 1.07. From (28), one gets for the relative norm,

(29) $$\lambda^{\prime }=\frac{\lambda }{\sqrt{q}},$$

the following inequality (30):

(30) $$\lambda^{\prime }\leq \alpha .$$

GLR fails in attacking the CPKC private key/message when (30) is not satisfied for the secret vector relative norm (f,g), i.e., if:

(31) $$\left||\left(f,g\right)|\right|/\sqrt{q}>\alpha$$

holds, GLR fails to find the CPKC private key/message.

CPKC selects small values for private key (f,g) in (7) to satisfy the decryption correctness condition (15). Hence, our goal is to propose in Section 5 a modification for CPKC, that is RCPKC, where (f,g) satisfies (31) and provides correct decryption for valid users and incorrect decryption for an attacker using GLR.

## 5. The Proposed RCPKC

In this section, random CPKC (RCPKC), an adjustment of CPKC described in Section 3.3, so that it becomes resistant to GLR attack, is proposed.

### 5.1. RCPKC’s Main Ideas

The main two ideas of RCPKC are:

- Contrary to the settings (7) of CPKC, which uses secret key (f,g) with a small norm not exceeding $\sqrt{q}$ so that (f,g) may be found as a shortest vector (SV) in the lattice $E(V_{1},V_{2})$ defined by (24), RCPKC [42] (we call it in this section RCPKC.1) was originally proposed having private key (f,g) with a large norm meeting (31) so that it cannot be returned by LBRA using GLR as the SV, but (f,g) also meets (15) due to the skew in its components.
- However, as mentioned in Section 4.1, for any pair of integers, *F* and *G*, satisfying (23), (F,G) is likely to serve as the first two components, f,g, of the private key. That means, in spite of the large norm of (f,g), the SV=(F,G), obtained in the result of LBRA using GLR, may meet the decryption correctness condition (15) and, thus, may be used for the correct plaintext message disclosure (Example S4 shows the LBRA attack using GLR against RCPKC.1; see the Supplementary Materials). That is why RCPKC.1, Section 5.2, before encrypting by (13) (contrary to CPKC using a random number from the predefined range (12)), defines a range for the random number selection using the SV, (F,G) (returned by GLR attack on the lattice $E(V_{1},V_{2})$ defined by (24)), so that the decryption correctness condition (15) holds for (f,g), but does not hold for (F,G), which leads to the failure of LBRA using GLR on RCPKC.1. Such an interval defined in (40)–(42) for RCPKC.1 is found to be vulnerable to the GLR attack. Therefore, an enhanced RCPKC proposed herein (we call it in this section RCPKC.2) with a tighter interval for *r* is defined in (46), (50) and (51), so that such an attack is inactive.

Thus, RCPKC.2 assumes that the private key owner selects a range for a random value, *r* (used in encryption (13)), based on the secret key, (f,g), and respective SV, (F,G), in the lattice, $E(V_{1},V_{2})$, defined by (24), guaranteeing correct decryption for a valid user and incorrect decryption for an attacker using GLR. Because of the special choice of the random value range, the proposed algorithm is called random CPKC (RCPKC). The problem for RCPKC is that the range of random numbers as so defined may be rather narrow, and thus, the security of RCPKC may suffer. However, as will be shown, the range is rather large and may significantly exceed the range for a secret message.

In Section 5.2, CPKC is modified to RCPKC.1 [42] so that the secret key, (f,g), meets (15) and (31). In Section 5.3, RCPKC.1 is further modified to RCPKC.2, so that it becomes immune to the LBRA attack.

### 5.2. RCPKC.1 Description

To meet (31), it is required that:

(32) $$f,r\geq \alpha \cdot \sqrt{q}.$$

The LBRA by GLR failure condition (31) holds if (32) is true since:

$$\frac{\left||(f,g)|\right|}{\sqrt{q}}=\frac{\sqrt{f^{2}+g^{2}}}{\sqrt{q}}=\frac{\sqrt{\alpha^{2}\cdot q+g^{2}}}{\sqrt{q}}>\alpha ,$$

$$\frac{\left||(r,e-m)|\right|}{\sqrt{q}}=\frac{\sqrt{r^{2}+{\left(e-m\right)}^{2}}}{\sqrt{q}}=\frac{\sqrt{\alpha^{2}\cdot q+{\left(e-m\right)}^{2}}}{\sqrt{q}}>\alpha ,$$

for *g*, e−m≠0. Condition (32), in RCPKC.1, substitutes the conditions (7) and (12) on *f* and *r* in CPKC. The message, *m*, and the private key, *g*, instead of (11) and (7), used in CPKC, are redefined in RCPKC.1 as follows:

(33) $$2^{mgLen}>g\geq 2^{mgLen-1}>m\geq 0,$$

where mgLen represents the length of *m* and *g* in bits.

For RCPKC.1, the correctness decryption condition (15) shall hold, which is true (see (39)) when the *f* and *r* values in addition to (32) meet (34):

(34) $$\frac{q}{2\cdot 2^{mgLen}}>f,r.$$

Since:

(35) $$q=2^{qLen},$$

then (32) and (34) can be rewritten:

(36) $$2^{qLen-mgLen-1}>f,r\geq \alpha \cdot 2^{qLen/2}.$$

To have a non-empty range for *f* and *r*, of a width of at least $\alpha \cdot 2^{qLen/2}$, from (36), the following condition is obtained:

(37) $$\frac{2^{qLen/2}}{2\cdot \alpha }>2^{mgLen+1}.$$

By defining $\beta =\log_{2}1/(2\cdot \alpha )\approx -1.103$, (37) shows that:

$$2^{\beta }\cdot 2^{qLen/2}>2^{mgLen+1},$$

qLen+2·β>2·(mgLen+1),

(38) qLen>2·(mgLen+1−β).

Let us show that the decryption correctness condition (15) holds when (33), (36) and (38) hold:

$$r\cdot g+f\cdot m<2^{qLen-mgLen-1}\cdot 2^{mgLen}+2^{qLen-mgLen-1}\cdot 2^{mgLen-1}$$

(39) $$<2^{qLen-1}+2^{qLen-1}=2^{qLen}=q.$$

Thus, for RCPKC.1, the norm of (f,g) meets (31), and the decryption correctness condition (39) holds. We need additionally that decryption correctness condition (39) to be violated for (F,G), that is the SV obtained in the result of the GLR attack on the lattice $E(V_{1},V_{2})$ defined by (24). Hence, it cannot be used as a private key for the plaintext message’s correct decryption.

Inequality (36) defines a range for *r* so that f,g,r,m meet (15). Now, we define constant on *r*,

(40) r≥rmin≥(q+g|F|)/|G|

such that F,G,r,m violate (15). Using (40) and (33):

(41) $$\begin{array}{cc} \left|G\cdot r+F\cdot m\right| & \geq \left|G\right|\cdot \left|r\right|-\left|F\right|\cdot m\geq \frac{\left|G|(q+g|F|\right)}{\left|G\right|}-\left|F\right|\cdot m\\ \; & \geq q+g|F|-m|F|>q. \end{array}$$

Thus, Inequality (36) is used for *f*, but for *r*, from (40) and (36), we have:

(42) $$2^{qLen-mgLen-1}>r\geq max\left(\alpha \cdot 2^{qLen/2},rmin\right).$$

For RCPKC security, the range defined by (42) shall be rather large, max($\alpha \cdot 2^{qLen/2},rmin$); hence:

(43) $$2^{qLen-mgLen-1}\geq 2\cdot max\left(\alpha \cdot 2^{qLen/2},rmin\right).$$

Thus, RCPKC.1 is defined as follows.

**RCPKC.1 definition:**

The private key components, (f,g), meet (8), (9), (33) and (34), where qLen, mgLen meet (38) and (43), where (F,G) is an SV obtained in the result of the GLR attack on the lattice E(V1,V2) defined by (24). The public key component, *h*, is defined by (10). Message, *m*, meets (33), and random integer, *r*, is selected from the range defined in (40) and (42). Encryption and decryption follow (13) and (14), (16), respectively (see Section 3.3.1 and Section 3.3.2). The decryption correctness condition (15) is proven for RCPKC.1 in (39). Examples S3 and S4 in the Supplementary Materials present an example of RCPKC.1 encryption/decryption and the LBRA attack using the GLR attack against RCPKC.1, respectively.

In the following section, RCPKC.1 is modified to RCPKC.2, so that it becomes immune against the LBRA attack.

### 5.3. RCPKC.2 Proposal

In order to resist the GLR attack against RCPKC.1, as shown in Example S3, the definition of the region from which *r* is selected should consider all SVs with a norm less than a threshold μ||(f,g)|| as follows.

The random interval defined in (40), (42) and (43) using only the SV obtained by the GLR attack on the lattice $E(V_{1},V_{2})$ defined by (24) must be modified to include all the SVs with a norm less than the norm of the secret key, by threshold μ||(f,g)|. Hence, all vectors $\left(F_{i},G_{i}\right)$ obtained in the course of GLR reduction that have norms:

(44) $$\left|\right|\left(F_{i},G_{i}\right)\left||<\mu |\right|\left(f,g\right)\left|\right|,i=1,\ldots ,N,$$

where *N* is the number of (F,G) pairs satisfying (44), μ is a threshold, e.g., μ=10, and then, it must be checked that:

(45) $$\left(\forall i=1,\ldots ,N\right)(\left(F_{i},G_{i}\right)\neq \left(f,g\right)).$$

If (45) is violated, i.e., one of the vectors in the list is our vector (f,g), then another (f,g) is used.

Inequality (36) defines a range for *r* so that f,g,r,m meet (15). Now, the constraint on *r* is defined as follows:

(46) $$q/g-f\geq rmax\geq r\geq rmin\geq (q+g\cdot \max_{i=1,\ldots ,N}|F_{i}\left|\right)/\min_{i=1,\ldots ,N}\left|G_{i}\right|,$$

such that $F_{j},G_{j},r,m$ violate (15) for any j=1,…,N. We require also that:

(47) h·rmin>q.

Using (33) and (46), it is noticed that actually, the decryption correctness condition (15) for any j=1,…,N, is violated:

(48) $$\begin{array}{ccc} |G_{j}\cdot r+F_{j}\cdot m| & \geq & |G_{j}\cdot r|-|F_{j}\cdot m|\geq |G_{j}|\cdot \frac{q+g\cdot \max_{i=1,\ldots ,N}\left|F_{i}\right|}{\min_{i=1,\ldots ,N}\left|G_{i}\right|}-\left|F_{j}\cdot m\right|\\ & \geq & q+g\cdot \max_{i=1,\ldots ,N}|F_{i}\left|-\right|F_{j}\cdot m|>q. \end{array}$$

From (33) and (46), it is also perceived that the decryption correctness condition (15) holds for the original (f,g):

(49) g·rmax+f·m≤g(q/g−f)+f·m=q−f·g+f·m<q

Thus, Inequality (36) is used for *f*, but for *r* from (36) and (46):

(50) $$rmax>r\geq max(\alpha \cdot 2^{qlen/2},rmin).$$

For RCPKC.2’s security, the range defined by (50) shall be rather large, such as, e.g., max($\alpha \cdot 2^{qlen/2},rmin$); hence, it is desirable to have:

(51) $$rmax\geq 2\cdot max(\alpha \cdot 2^{qlen/2},rmin).$$

In order to provide CCA indistinguishability (see Definition 2 and Section 7), it is required to have:

(52) gcd(g,q)>1.

Thus, the RCPKC.2 proposal follows.

**RCPKC.2 proposal:**

The private key components, (f,g), meet (8), (9), (33), (34) and (52), where qLen, mgLen meet (38) and (43) The public key component, *h*, is defined by (10). Message, *m*, meets (33), and random integer, *r*, is selected from the range defined in (46), (47) and (50). Encryption and decryption follow (13), (14) and (16), respectively (see Section 3.3.1 and Section 3.3.2). The decryption correctness condition (15) is proven for RCPKC in (49). Example S5 in the Supplementary Materials presents an example of finding RCPKC.2’s random interval and LBRA by GLR failure.

RCPKC.2 is more secure than RCPKC.1 because intermediate GLR outputs are also used for the random parameter range selection. However, their computational complexity is the same, since both employ GLR and follow the same encryption/decryption procedures.

RCPKC.2 is also resistant to various attacks, as shown in the security analysis presented in the next section. Note that hereafter, RCPKC.2 is again denoted as RCPKC.

## 6. RCPKC Security Analysis

In this section, attacks on NTRU are considered (brute force (on the key and message), meet-in-the-middle (MITM) in Section 6.1, lattice basis reduction in Section 6.4, hybrid lattice basis reduction and MITM [52] in Section 6.2, multiple transmission (MTA) [11] in Section 6.3, and also, the most recent, chosen ciphertext [53,54,55,56], in Section 6.5), and we try applying them to RCPKC. Herein, the NTRU parameters used, EES401EP1 [41], of the security level, k=112 bits:

$$N=401,p=3,q=2048,df_{1}=df_{2}=8,$$

(53) $$df_{3}=6,dg=133,dr_{1}=dr_{2}=8,dr_{3}=6.$$

In order to meet the same security level, the RCPKC settings satisfying (38) are:

(54) qLen=473,mgLen=225.

The key space cardinality (defined in Section 6.1 for the parameters (53) and (54)) is greater than or equal to $2^{2\cdot k}$ for *k* = 112 to avoid the MITM attack explained in Section 6.1.

### 6.1. Brute Force and MITM Attacks

An attacker can recover the NTRU private key by trying all possible values of *g* and testing whether f·hmodq has small coefficients (the product corresponds to *g* according to (10)). On the other hand, an attacker can try all possible values of *g* and test whether $h^{-1}\cdot g\;\mathrm{mod}\;q$ (corresponding to *f* by virtue of (10)) has small coefficients. Equations (55) and (56) show the search space cardinalities for *g* and *f* for the security level, *k* = 112 (taking into account the MITM attack explained later in this section). The search space cardinality for *f* is computed as follows (see [53] (Section 7)):

(55) CNTRU(f,k)=Ndf1N−df1df1Ndf2N−df2df2Ndf3N−df3df3=401839384018393840163956=1.16×1090≥22·k=2224.

Similarly, for *g*:

(56) CNTRU(g,k)=NdgN−dgdg=401133268133=4.34×10188≥22·k=2224.

it is perceived the search space cardinality for *f* is less than that for *g*, so the best strategy for an attacker is to search for *f* values.

An attacker can reduce the search space cardinality from $2^{k}$ to $2^{k/2}$ [57] using MITM by splitting the private key *f* (which is a polynomial of degree N−1) into two polynomials, $f=f_{1}+f_{2}$, where $f_{1}$ is a polynomial of degree at most N/2−1 and polynomial $f_{2}$ contains terms of degree between N/2 and N−1, and then trying matches: $f_{1}\cdot h\;\mathrm{mod}\;q=\left(g-f_{2}\cdot h\right)\;\mathrm{mod}\;q.$ Hence, in order to meet the *k* = 112 security level, the NTRU parameters must be chosen to meet the *k* = 224 security level, as it is already made in (53). For RCPKC, the secret value, *g*, is selected from the interval $\left[2^{mgLen-1},2^{mgLen}\right)$ (see (33)); hence, the search space cardinality for *g* to meet the 2·k-bit security level against the brute force attack shall satisfy:

(57) $$C_{RCPKC}\left(g,k\right)=2^{mgLen-1}\geq 2^{2\cdot k}.$$

The secret value, *f*, is selected from the interval $\left[\alpha \cdot 2^{qLen/2},2^{qLen-mgLen-1}\right)$ (see (36)); hence, the search space cardinality for *f* to meet the 2·k-bit security level against the brute force attack shall satisfy:

(58) $$C_{RCPKC}\left(f,k\right)=2^{qLen-mgLen-1}-\alpha \cdot 2^{qLen/2}\geq 2^{2\cdot k}.$$

For the parameters (54), $C_{RCPKC}\left(g,k\right)=2^{224}$, while $C_{RCPKC}\left(f,k\right)\approx 2^{247}$. In order to provide the security level for k=112, the parameters (54) are chosen to meet the twice greater security level of 2·k=224 to counter the MITM attack, considered below, which reduces the brute force attack effort by the square root. Since $C_{RCPKC}\left(g,k\right)<C_{RCPKC}\left(f,k\right),$ the best strategy for an attacker is to search for *g* values. Similar to NTRU, the MITM attack can be applied to the RCPKC private key component, *g*. Since mgLen is the bit length of *g*, then $g=g_{1}+2^{(mgLen-1)/2}g_{2}$, and then, $g_{1}$ and $g_{2}$, each of a bit length equal to (mgLen−1)/2, can be enumerated with the resulting search space cardinality $O(2^{(mgLen-1)/2})$ trying to find matching:

$$\left(f\cdot h-g_{1}\right)\;\mathrm{mod}\;q=2^{(mgLen-1)/2}g_{2}\;\mathrm{mod}\;q.$$

Thus, the RCPKC parameters (54) provide the security level *k* = 112 against the brute force attack with MITM. Now, let us consider the brute force attack on the message.

An attacker can compromise an NTRU message by trying all possible values of *r* and testing whether e−r·hmodq has small coefficients. Similarly, the attacker can compromise the RCPKC message by trying all possible values of *r* and testing if $e-r\cdot h\;\mathrm{mod}\;q\in [0,2^{mgLen-1})$ by virtue of (33).

The RCPKC message search space is defined by the interval $\left[0,2^{mgLen-1}\right)$ (see (33)); hence, the search space cardinality for *m* to meet the 2·k-bit security level against the brute force attack shall satisfy:

(59) $$C_{RCPKC}\left(m,k\right)=2^{mgLen-1}\geq 2^{2\cdot k},$$

while the search space of *r* is defined by (46), (50) and (51). Hence, the search space cardinality for *r* to meet the 2·k-bit security level against the brute force attack shall satisfy:

(60) $$C_{RCPKC}\left(r,k\right)=rmax-max\left(\alpha \cdot 2^{qLen/2},rmin\right)\geq 2^{2\cdot k}.$$

Table 1 shows the mgLen and qLen values to meet different 2·k-bit security levels’ condition (60) (see Rows 1 and 2) and the width of the range for *r* (Row 7) with *f* and *g* specified in Rows 3 and 4, respectively. It proves that the method can be practically used.

### 6.2. A Hybrid Lattice Basis Reduction and MITM Attack

The attack [52] on the NTRU secret key combines the LBRA and MITM strategies. The hybrid attack, first, splits the original lattice of order 2N, N>1, into three subparts, only one of which is further reduced, whereas the vectors from the other parts are just enumerated, thus combining the concepts of the LBRA and MITM attacks. The hybrid attack is not applicable to RCPKC since:

- The RCPKC lattice is two-dimensional and cannot be split into the three subparts;
- RCPKC uses a large norm secret (f,g) vector (see (33) and (36)) that cannot be found by LBRA looking for an SV, and the SV cannot be used for correct decryption (see (48)).

### 6.3. Multiple Transmission Attack

MTA reveals a large part of an NTRU message by sending *n* times one and the same message, *m*, using the same public key, *h*, but different random values, $r^{i}$. For NTRU encryption (13) (see Section 3.1):

$$e^{i}=r^{i}\cdot h+m\;\mathrm{mod}\;q$$

for i=1,2,…,n. An adversary computes:

$$\left(e^{i}-e^{1}\right)\cdot h^{-1}\;\mathrm{mod}\;q,$$

thereby recovering $r^{i}-r^{1}\;\mathrm{mod}\;q,$i=1,…,n, and from these relations, many coefficients of $r^{1}$ may be revealed. Knowledge of $r^{1}$ allows disclosing the message, *m*. RCPKC is not susceptible to MTA because no special structure is assumed for $r^{1}$ contrary to the case of NTRU.

### 6.4. Lattice Basis Reduction Attacks

The NTRU lattice basis, $L_{h}^{NTRU}$, associated with public key *h* defined in (1) is:

$$L_{h}^{NTRU}=\left(\begin{array}{cccccccc} 1 & 0 & \ldots & 0 & h_{0} & h_{1} & \ldots & h_{N-1}\\ 0 & 1 & \ldots & 0 & h_{N-1} & h_{0} & \ldots & h_{N-2}\\ \vdots & \vdots & \ddots & \vdots & \vdots & \vdots & \ddots & \vdots \\ 0 & 0 & \ldots & 1 & h_{1} & h_{2} & \ldots & h_{0}\\ 0 & 0 & \ldots & 0 & q & 0 & \ldots & 0\\ 0 & 0 & \ldots & 0 & 0 & q & \ldots & 0\\ \vdots & \vdots & \ddots & \vdots & \vdots & \vdots & \ddots & \vdots \\ 0 & 0 & \ldots & 0 & 0 & 0 & \ldots & q \end{array}\right),$$

where $h_{0},\ldots ,h_{N-1}$ are the coefficients of the polynomial *h*. For convenience, matrix $L_{h}^{NTRU}$ is abbreviated as:

$$L_{h}^{NTRU}=\left(\begin{array}{cc} I & h\\ 0 & qI \end{array}\right).$$

The NTRU private key recovery problem can be formulated as the SVP in 2N-dimensional lattice, $L_{h}^{NTRU}$. Actually, if a polynomial, *b*, of degree N−1 with integer coefficients satisfying:

f·h+q·b=g

exists, then:

$$\left(f,b\right)\cdot L_{h}^{NTRU}=\left(f,g\right).$$

Therefore, the vector (f,g) is in the lattice $L_{h}^{NTRU}$. Vector (f,g) or its rotation (rotation of a polynomial, *f*, by *i* steps is $x^{i}\cdot f\in R_{q}$ for an integer *i*) can be found if it is the shortest vector in $L_{h}^{NTRU}$. The lattice reduction algorithm LLL [51] finds the shortest vector in $L_{h}^{NTRU}$ in time exponential in *N*. According to [40], LLL takes 1.05 $\times 10^{31}$ MIPS (million instructions per second)-years to find the shortest vector or its rotation for *N* = 400 (as in (53)) that most likely is the NTRU private key part, (f,g).

Contrary to NTRU, RCPKC is resistant to LBRA since the GLR attack fails for it (see Section 5). LBRA is one of the most used and effective techniques in attacking an NTRU private key (e.g., it is used in the hybrid lattice attack, the most efficient on practical NTRU parameters [58]; see Section 6.2), but it is not applicable to RCPKC.

### 6.5. Chosen Ciphertext Attack

Three chosen ciphertext attacks (CCA) on NTRU are known. The first key recovering CCA described in [54] uses a ciphertext of a special shape, which can be countered by message padding [53]. Standardized parameters [53] allow decryption failure, i.e., a ciphertext could fail to be decrypted correctly by NTRU. In [55], a CCA was presented where an attacker collects a large number of decryption failures; see the NTRU correction decryption condition (5) in Section 3.1. Another CCA was presented in [56], which is more efficient than [55], but still depends on decryption failures. RCPKC works on non-structured integers, and the parameters, set in Section 5, guarantee correct decryption. Thus, neither of the CCAs described above are applicable to RCPKC.

## 7. RCPKC Asymmetric Encryption Padding and its IND-CCA2 Security

In this section, we prove the security of the RCPKC one-way function based on the discussions of the security of the NTRU one-way function in [8], define RCPKC asymmetric encryption padding (RAEP), and prove its IND-CCA2 security as a particular case of NAEP. According to Section 5.2 and Section 5.3, RCPKC defines the following four sets:

- $D_{f}=\left[\alpha \cdot 2^{qLen/2},2^{qLen-mgLen-1}\right)$: private key space, an interval from which a private key, *f*, is selected;
- $D_{g}=\left[2^{mgLen-1},2^{mgLen}\right)$: private key space, an interval from which a private key, *g*, is selected;
- $D_{m}=\left[0,2^{mgLen-1}\right)$: RCPKC plaintext space, an interval from which a plaintext, *m*, is selected;
- $D_{r}=\left[max\left(\alpha \cdot 2^{qLen/2},rmin\right),rmax\right]$: RCPKC random value space.

The RCPKC encryption primitive is specified by the parameter set, $P=(q,D_{f},D_{g},D_{m},D_{r})$. The one-way function underlying RCPKC is:

$$F_{h}:D_{m}\times D_{r}\to Z_{q},$$

$$F_{h}\left(m,r\right)=r\cdot h+m\;\mathrm{mod}\;q.$$

**Definition** **3.** *RCPKC-OW problem: For a parameter set, P, we denote by $Succ_{RCPKC}^{OW}\left(A,P\right)$ the success probability of a PPT adversary, A, for finding a pre-image of $F_{h}$,*

$$Succ_{RCPKC}^{OW}\left(A,P\right)=\mathrm{Pr}\left(\begin{array}{c} \left(m^{\prime },r^{\prime }\right)\leftarrow A\left(e,h\right)\\ s.t.\left(\exists r^{\prime }\in D_{r}\right)\;\left(F_{h}\left(m^{\prime },r^{\prime }\right)=e\right) \end{array}\right).$$

**Assumption** **2.** *RCPKC-OW assumption: For every PPT adversary, A, solving the RCPKC-OW problem, there exists a negligible function, $v_{A}\left(k\right)$, such that for sufficiently large k, we have:*

$$Succ_{RCPKC}^{OW}\left(A,P\right)\leq v_{A}\left(k\right).$$

An adversary $A_{1}$ can compromise (m,r) by picking $r^{\prime }\in D_{r}$, substituting it in $(e-r^{\prime }\cdot h)\;\mathrm{mod}\;q$, and checking, if the result is in $D_{m}$. Thus, $Succ_{RCPKC}^{OW}\left(A_{1},P\right)$ is:

$$Succ_{RCPKC}^{OW}\left(A_{1},P\right)=\frac{2^{mgLen}}{2^{qLen}}.$$

Since qLen>mgLen by definition (38), $Succ_{RCPKC}^{OW}\left(A_{1},P\right)$ decreases exponentially in qLen, and Assumption 1 holds. Similarly, the attacker can try the following methods with an exponentially decreasing success probability:

1. The adversary, $A_{2}$, chooses randomly a pair $\left(r^{\prime }\in D_{r},m^{\prime }\in D_{m}\right)$ and checks if $r^{\prime }\cdot h+m^{\prime }\;\mathrm{mod}\;q=e$.
2. The adversary, $A_{3}$, picks $f^{\prime }\in D_{f}$, substitutes it in $f^{\prime }\cdot h\;\mathrm{mod}\;q$, and checks whether the result is in $D_{g}$.
3. The adversary, $A_{4}$, chooses randomly a pair $\left(f^{\prime }\in D_{f},g^{\prime }\in D_{g}\right)$, if possible, calculates $h^{\prime }$, decrypts *e* to $\left(r^{\prime },m^{\prime }\right)$, and checks if $r^{\prime }\cdot h^{\prime }+m^{\prime }\;\mathrm{mod}\;q=e$.
4. Furthermore, the adversary can apply the GLR attack to get (f,g). However, by construction, RCPKC is immune to that attack, and hence, the success probability is zero.
  Therefore, Assumption 1 is true for all the above attacks.

RCPKC encryption (13) differs from NTRU encryption (2) just by setting N=p=1. The conclusion of [7] on NAEP IND-CCA2 security is also true for asymmetric encryption padding, RAEP. However, NAEP cannot be used as is for N=p=1 because it utilizes specific true polynomial functions center() and compress(). Since the decryption correctness condition (15) holds for RCPKC due to the parameter choice, the center() function is not used in RCPKC and RAEP. The function compress() as in NAEP shall map its input, p·r·h, to a binary string, bs, of the padded message size. In NAEP, it is done in two steps: s=compress(p·r·hmodq);bs=H(s). In RAEP, both transforms are done by one hash function, $H:Z_{q}\to {\left\{0,1\right\}}^{mgLen}$. Algorithms 3 and 4 show RAEP encryption and decryption, respectively.
**Algorithm 3:** RAEP encryption.
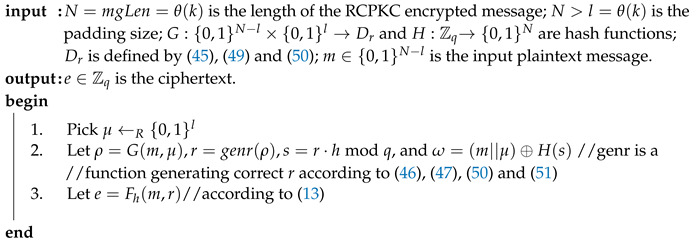


**Algorithm 4:** RAEP decryption.

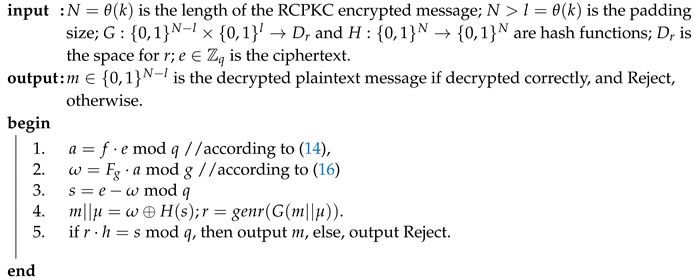



## 8. RCPKC Performance and Power Consumption Evaluation

### 8.1. RCPKC Performance Evaluation

Experiments were conducted using the NTRU code [59] and RCPKC implementation in the C99 language similar to [59] with the NTL library [60] on a PC equipped with 1.6 GHz Intel Core i5-8250U, 8 GB RAM, and Windows 10 (see Tables S1 and S2 of the Supplementary Material for the RCPKC performance experiments’ results and the NTRU performance experiments’ results, respectively; the RCPKC source code is available in [61]). Both the NTRU code [59] and the proposed RCPKC were implemented in Visual Studio 2017. The NTRU parameters (53) and the RCPKC parameters (54) were used. The CPU encryption and decryption time of RCPKC and NTRU was measured for $10^{3},10^{4},$ and $10^{5}$ runs. In each run, a distinct 128 bit message was encrypted/decrypted with both cryptosystems. The NTL function RandomLen() was used to pseudo-randomly generate the messages. RandomLen() was seeded with the output of the function clock(). The generated messages were stored in a separate file and used to test RCPKC and NTRU. The CPU time was measured via QueryPerformanceCounter() with ns accuracy. Table 2 shows the sample mean, $\bar{x}$, standard deviation, σ, and confidence interval with the confidence level C∈{0.95,0.99,.0.999} for the number of runs $n\in \{10^{3},10^{4},10^{5}\}$, respectively for RCPKC and NTRU. The confidence interval, [l,u], is calculated using [62] (p. 358):

(61) $$\left[l,u\right]=\left[\bar{x}-z^{*}\frac{\sigma }{\sqrt{n}},\bar{x}+z^{*}\frac{\sigma }{\sqrt{n}}\right],$$

where $\bar{x}=\frac{\sum_{i=1}^{n}x_{i}}{n},\sigma =\frac{\sqrt{\sum_{i=1}^{n}{\left(x_{i}-\bar{x}\right)}^{2}}}{n-1},x_{i},$ and *n* are the sample mean, sample standard deviation, value of the run, and number of runs, respectively; $z^{*}$ is the critical value required for the specific confidence level; see Table C in [62] (p. 746). For example, in Table 2 for RCPKC encryption with C=95%, $n=10^{3},\bar{x}=6.19\times 10^{-6},\sigma =3.966\times 10^{-6},z^{*}=1.960$, the confidence interval is calculated as follows: $\left[l,u\right]=\left(6.190\times 10^{-6}-1.960\left(3.966\times 10^{-6}\right)/\sqrt{1000},6.190\times 10^{-6}+1.960\left(3.966\times 10^{-6}\right)/\sqrt{1000}\right)=\left(6.112\times 10^{-6},6.267\times 10^{-6}\right)$.

**Figure 1:**
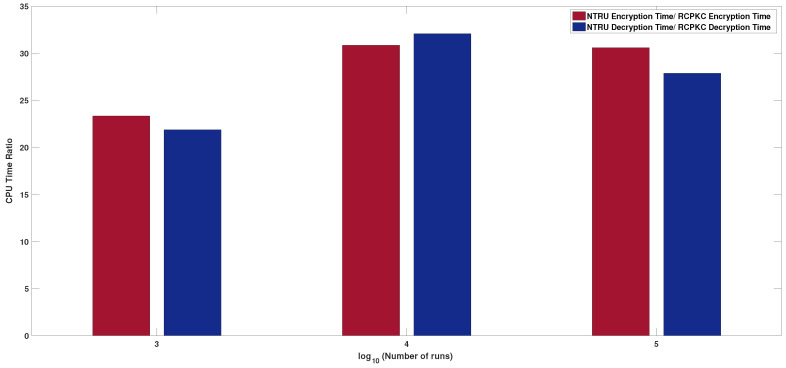
NTRU/RCPKC encryption and decryption average CPU time ratio for $10^{3},10^{4},$ and $10^{5}$ runs.

Figure 1 shows the NTRU/RCPKC encryption and decryption average CPU time ratio for $10^{3},10^{4},$ and $10^{5}$ runs. From Figure 1, it is observed that RCPKC is 27.08±3.75 times faster than NTRU in encryption and 26.9±5.09 times faster in decryption, respectively. Table 3 compares NTRU versus RCPKC and several NTRU variants presented in Section 1. It is observed that RCPKC is faster than the fastest most recently published NTRU variant, BQTRU, more than four times in encryption.

### 8.2. RCPKC Power Consumption Evaluation

In this section, RCPKC’s power consumption is compared to NTRU in two cases: applying both algorithms using the same or different frequencies.

**Same frequencies**: Let the RCPKC and NTRU execution time be $T_{RCPKC}$ and $T_{NTRU}$, respectively. Then, from (18), the consumed energy by NTRU and RCPKC $E_{NTRU}$ and $E_{RCPKC}$ is:

(62) $$\begin{array}{cc} E_{NTRU} & =P\cdot T_{NTRU},\\ E_{RCPKC} & =P\cdot T_{RCPKC}. \end{array}$$

Since $T_{NTRU}$ is greater than $T_{RCPKC}$ by more than 27 times, then from (62):

(63) $$\frac{E_{NTRU}}{E_{RCPKC}}=\frac{T_{NTRU}}{T_{RCPKC}}\geq 27.$$

From (63), RCPKC consumes twenty seven times less energy than NTRU using the same frequency.

**Different frequencies**: Since RCPKC is 27 times faster than NTRU, the former takes approximately the same run time on a 27 times lower clock frequency CPU than that of the latter. Dynamic and leakage power consumption, calculated for frequencies from [48] (p. 19) according to (21), are shown in Table 4.

It follows from Table 4 that $P_{leak}\ll P_{dyn}$, and it can be neglected. From Table 4, it follows that reducing the clock frequency from 16 to 1 MHz leads to a 16 times power consumption reduction from 1440 to 90 μW. Note that MSP430FR5969, at a lower frequency, operates at a lower voltage: operating on a 1 MHz frequency at 2.2 V [48] (p. 19) results in 48.4 μW of dynamic power consumption. Hence, the total power reduction is $\frac{1440}{48.4}\approx 30$ times. Therefore, RCPKC, compared to NTRU, is better applicable to WSNs with power constrained devices.

## 9. Conclusions

In this paper, RCPKC is proposed, a secure and effective congruential, modulo *q*, public-key cryptosystem using big numbers. It uses the same encryption/decryption mechanism as NTRU does, but works with numbers. Contrary to NTRU, RCPKC is resistant to LBRA because its private key components, *f* and *g*, are chosen big with respect to $\sqrt{q}$ to form a two-component vector with the norm exceeding Minkowski’s boundary (27)–(30) for the shortest vector in a two-dimensional lattice and meeting (31). Hence, LBRA by the GLR algorithm returning the shortest vector in a two-dimensional lattice fails at finding the large norm private key vector, (f,g).

In spite of the big numbers, *f* and *r*, meeting (36) used in RCPKC, it guarantees that the decryption correctness condition (15) holds (see (39)) due to the use of Conditions (33), (36), (38), (46) and (50) instead of Conditions (7), (11), and (12), used in the original insecure CPKC (see Section 3.3, Section 3.3.1, Section 3.3.2) considered in [26]. It was found that the insecurity of the original CPKC stems from the use of Conditions (7), (11) and (12), defining smaller than $\sqrt{q}$ numbers *f*, *g*, *m*, *r* meeting Minkowski’s boundary (27) and the decryption correctness condition (15). RCPKC is resistant to the LBRA by GLR attack due to the special choice of the range for the random value, *r*, used in the encryption (13) that guarantees correctness condition (15) violation for the short vectors returned by GLR, but holding for the original private key, (f,g). Section 6 shows also that the security of RCPKC with respect to other known attacks on NTRU is not less than that of NTRU, which allows us to conclude that RCPKC is more secure than NTRU. Section 7 proves the IND-CCA2 security of RCPKC asymmetric encryption padding (RAEP).

RCPKC uses numbers, i.e., minimal possible, degree zero, polynomials, which makes it about 27 times more effective in encryption and decryption than NTRU and more than three times more effective in encryption with respect to the fastest most recently published NTRU variant, BQTRU [21], as the experiments show (see Table 3). Compared to NTRU, RCPKC reduces the energy consumption at least 27 times, which allows increasing the life-time of unattended WSNs by more than 27 times.

As a future work, the proposed RCPKC will be applied to telemedicine to secure the data collected by medical sensors and cameras. 

## Figures and Tables

**Table 1:** Width of the range for the *r* value for different security levels (Row 7); the parameters of the random congruential public key cryptosystem (RCPKC) affecting the width ($mgLen,qLen,f,g,rmax,max(\alpha \cdot 2^{qLen/2},rmin)$) are specified in Rows 1–6.

#	RCPKC Parameter	2×k		
		224	336	448
1	mgLen	225	337	450
2	qLen	473	743	909
3	$f=2^{qLen-mgLen-1}-1$	$2.26\times 10^{74}$	$8.26\times 10^{121}$	$7.44\times 10^{137}$
4	*g*	$2^{mgLen}-1=$	$2^{mgLen}-5=$	$2^{mgLen}-11=$
		$5.39\times 10^{67}$	$2.79\times 10^{101}$	$2.90\times 10^{135}$
5	rmax	$2.26\times 10^{74}$	$8.26\times 10^{121}$	$7.44\times 10^{137}$
6	$max(\alpha \cdot 2^{qLen/2},rmin)$	$7.41\times 10^{72}$	$1.62\times 10^{119}$	$1.10\times 10^{137}$
7	$C_{RCPKC}\left(r,k\right)$	$2.1\times 10^{74}$	$8.24\times 10^{121}$	$6.34\times 10^{137}$

**Table 2:** RCPKC and NTRU CPU encryption/decryption time sample mean, standard deviation, and confidence interval for $10^{3},10^{4},$ and $10^{5}$ runs.

Algorithm	Measured Value	Run Number, *n*		
		$10^{3}$	$10^{4}$	$10^{5}$
	C	0.95	0.99	0.999
	$z^{*}$	1.960	2.576	3.291
RCPKC (Encryption)	Sample Mean, $\bar{x}$	$6.190\times 10^{-6}$	$5.492\times 10^{-6}$	$4.708\times 10^{-6}$
	Sample Standard			
	deviation, σ	$3.966\times 10^{-6}$	$2.076\times 10^{-6}$	$2.923\times 10^{-6}$
	Confidence	($6.112\times 10^{-6},$	($5.475\times 10^{-6},$	($4.677\times 10^{-6},$
	Interval, [l,u]	$6.267\times 10^{-6}$)	$5.508\times 10^{-6}$)	$4.738\times 10^{-6})$
NTRU (Encryption)	Sample Mean, $\bar{x}$	$1.444\times 10^{-4}$	$1.964\times 10^{-4}$	$1.440\times 10^{-4}$
	Sample Standard			
	deviation, σ	$6.878\times 10^{-5}$	$1.123\times 10^{-5}$	$6.437\times 10^{-5}$
	Confidence	($1.430\times 10^{-4}$,	($1.430\times 10^{-4}$,	($1.430\times 10^{-4}$,
	Interval, [l,u]	$1.457\times 10^{-4}$)	$1.973\times 10^{-4}$)	$1.447\times 10^{-4}$)
RCPKC (Decryption)	Sample Mean, $\bar{x}$	$9.506\times 10^{-6}$	$8.812\times 10^{-6}$	$7.493\times 10^{-6}$
	Sample Standard			
	deviation, σ	$2.781\times 10^{-6}$	$2.370\times 10^{-6}$	$2.795\times 10^{-6}$
	Confidence	($9.451\times 10^{-6}$,	($8.792\times 10^{-6}$,	($7.464\times 10^{-6}$,
	Interval, [l,u]	$9.560\times 10^{-6}$)	$8.831\times 10^{-6}$)	$7.522\times 10^{-6}$)
NTRU (Decryption)	Sample Mean, $\bar{x}$	$2.079\times 10^{-4}$	$2.826\times 10^{-4}$	$2.088\times 10^{-4}$
	Sample Standard			
	deviation, σ	$9.700\times 10^{-5}$	$1.594\times 10^{-4}$	$8.633\times 10^{-5}$
	Confidence	($2.060\times 10^{-4}$,	($2.813\times 10^{-4}$,	($2.079\times 10^{-4}$,
	Interval, [l,u]	$2.098\times 10^{-4})$	$2.839\times 10^{-4})$	$2.097\times 10^{-4})$

**Table 3:** Ratios of encryption and decryption times, $\gamma_{A}=\frac{T_{NTRU}^{ENC}}{T_{A}^{ENC}}$, $\delta_{A}=\frac{T_{NTRU}^{DEC}}{T_{A}^{DEC}}$, of NTRU and the algorithms A∈ {RCPKC, BQTRU, MaTRU, ETRU}.

Algorithm, *A*	Encryption Times Ratio, $\gamma_{A}$	Decryption Times Ratio, $\delta_{A}$
Proposed RCPKC	27	27
BQTRU [21]	7	No data
MaTRU [23]	2.5	2.5
ETRU [27]	1.45	1.72

**Table 4:** MSP430FR5969 microcontroller dynamic and leakage power consumption, $P_{dyn}$ and $P_{leak}$, for frequencies from [48] (p. 19) at active supply voltages of 3 and 2.2 V.

	2.2 V		3 V	
Frequency (MHz)	$P_{\mathrm{dyn}}$ (μW)	$P_{\mathrm{leak}}$ (nW)	$P_{\mathrm{dyn}}$ (μW)	$P_{\mathrm{leak}}$ (nW)
1	48.4	44	90	60
16	774.4		1440	

## Supplementary Materials

The following are available online at https://www.mdpi.com/1424-8220/20/16/4632/s1: Code S1: Maple code of LBRA by GLR, Table S1: RCPKC performance experiments’ results, Table S2: NTRU performance experiments’ results, Example S1: Example of CPKC encryption/decryption, Example S2: LBRA attack using GLR against CPKC, Example S3: Example of RCPKC.1 encryption/decryption, Example S4: LBRA attack using GLR against RCPKC.1, Example S5: Example of finding RCPKC.2’s random interval and LBRA by GLR failure.
