# Supplementary material for: NTRU-Like Random Congruential Public-Key Cryptosystem for Wireless Sensor Networks
Source: Sensors (Basel). 2020 Aug 17;20(16):4632. doi: 10.3390/s20164632 (PMC7472001; doi:10.3390/s20164632)
Supplement: Supplementary file 1 [file sensors-20-04632-s001.zip › Supplementary Material/Supplementary Material.pdf]

Supplementary Materials: NTRU-Like Random Congruential Public-Key Cryptosystem for Wireless Sensor Networks

Anas Ibrahim[1,2*], Alexander Chefranov[2], Nagham Hamad[1], Yousef-Awwad Daraghmi[1], Ahmad Al-Khasawneh[3], Joel J. P. C. Rodrigues[4,5]

1. Examples

Example S1. *Example of CPKC Encryption/ Decryption*

The example is close to Example 7.1, from [1, p. 375]. Maple code is available in Example S1 of the supplementary material.

Key Creation

Let according to (7), (8), $q = 122430513839$, $f = 231233$, and $g = 195696$. According to (9), $F_g = 127505$, and $F_q = 54368439252$ as shown in (1) and (2), Figure S1 Public key component, h, is calculated by (10) as shown in (3) of Figure S1 :

$$h = F_q \cdot g \bmod q = 107143708775.$$

Public key is (h, q), and private key is (f, g).

Encryption

Let according to (11) and(12), $r = 10101$ and $m = 12345$. The ciphertext, e as showin in (4) of Figure S1, is computed according to (13):

$$e = r \cdot h + m \bmod q = 95290525699.$$

In **Step 1** of the decryption process, equation (14) is applied as shown in (5) of Figure S1:

$$a = f \cdot e \bmod q = r \cdot g + f \cdot m = 4831296681.$$

In **Step 2**, the message m is retrieved using (16) as shown in (6), Figure S1:

$$m = F_g \cdot a \bmod g = 12345.$$

Thus, the plaintext $m = 12345$ is revealed. It can be seen that CPKC encryption/decryption procedure (13), (14), and (16), works correctly due to (15) holding.

Example S2. *LBRA attack using GLR against CPKC*

In this example, we try LBRA by GLR using code S1 on CPKC private key/message for the data from the Example S1. Maple code is available in Example S2 of the supplementary material. LBRA by GLR finds in 9 iterations the shortest vector, $v1$ = (231233, 195696) as shown in Figure S1. The shortest vector, $v1$, found by GLR corresponds to the private key components, (f, g), because they were selected small, having order $\mathcal{O}(\sqrt{q})$ values according to (7). Note that the norm of the vector, $(f, g) = \sqrt{f^2 + g^2} = 3.029284151 \times 10^5$ is small compared to $\sqrt{q} = 3.499007199 \times 10^5$. The message related vector, $(r, e - m)$, is not disclosed in the attack because $e = \mathcal{O}(q)$.

```
q := 122430513839 :
f := 231233 :
g := 195696 :

igcdex(f, q,'Fq','qz') :
igcdex(f, g,'Fg','gz') :
Fq mod q
                                54368439252                         (1)
Fg mod g
                                127505                              (2)
h := Fq·g mod q;
                                107143708775                        (3)
r := 10101 :
m := 12345 :
e := r·h + m mod q
                                95290525699                         (4)
a := f·e mod q
                                4831296681                          (5)
Fg·a mod g
                                12345                               (6)
GLR(f, g, h, q) :
                                "keys are found"
                                "keys are found"                    (7)
v1
                                [ 231233 ]
                                [ 195696 ]                          (8)
counter
                                9                                   (9)
```

Figure S1. Screenshot of LBRA by GLR using Maple Code S1 on CPKC for the data from the Example S1 finding the private key components, $(f, g) = v1$, in 9 iterations.

Example S3. *Example of RCPKC.1 Encryption/ Decryption*

In the following example, RCPKC.1 encryption/ decryption process is shown. Maple code of this example is available in Example S3 of the supplementary material.

Key Creation

Let $mgLen = 16$, $qLen = 80$, meeting (38), $q = 2^{qLen}$, private key component, $g = 2^{16} - 1$, is selected to meet (33). On the other hand, private key component, $f = 1{,}351{,}417{,}702{,}001$, is selected to meet (36). See (1)-(6) in Figure S2. According to (9), F_q, and F_g are calculated in (8) of Figure S2. Then, public key component, h, is computed using (10) as shown in (9) of Figure S2. RCPKC.1 public key is (h, q), and the private key is (f, g).

Encryption

To compute the interval from which r is selected (42) and (43), lower boundary is found in (10)-(15) in Figure S2. To encrypt message $m = 14$, random number $r = 1{,}176{,}477{,}442{,}250$ is selected from the interval. Then, ciphertext e, is calculated using (13) as shown in (18) of Figure S2.

Decryption

For decryption, in the first step, according to (14), we find a by as the product of the ciphertext, e, and the private key f modulo q as shown in (19) of Figure S2. In the second decryption step, according to (16), we multiply, a, by F_g to get the message m as we can see in (20) of Figure S2. Hence, the message, m, is correctly retrieved.

Settings of RCPKC−1

$mgLen := 16 :$
$m := 14 :$
$qLen := 80 :$
$q := 2^{qLen};$

$$1208925819614629174706176 \quad (1)$$

$g := 2^{16} - 1 :$
$is(\ g < 2^{mgLen}\ \mathbf{and}\ g \geq 2^{mgLen-1});$

$$true \quad (2)$$

Lower boundary for f

$\mathrm{alpha} := 1.07 :$
$lb := \mathrm{ceil}(\mathrm{alpha} \cdot \mathrm{sqrt}(q)) ;$

$$1176477442000 \quad (3)$$

Upper boundary for f, r

$Ub := 2^{qLen - mgLen - 1};$

$$9223372036854775808 \quad (4)$$

$f := 1351417702001;$

$$1351417702001 \quad (5)$$

$is(f < Ub\ \mathbf{and}\ f \geq lb);$

$$true \quad (6)$$

$gcd(f, q \cdot g) ;$

$$1 \quad (7)$$

Computing inverse of f modulo q, g

$igcdex(f, q, 'Fq', 'qz') :$
$igcdex(f, g, 'Fg', 'gz') :$
$Fq;$
$Fg;$

$$15426040477058097907982 5$$
$$2291 \quad (8)$$

Computing public key h

$h := Fq \cdot g\ \mathbf{mod}\ q;$

$$417923022495305103287663 \quad (9)$$

Applying GLR to set lower boundary for r

$GLR(f, g, h, q)$

$$\begin{bmatrix} 1133958117 & 459459339518 & 26960316553 & 459459339518 & 891958362483 \\ 44728059201205 & 894561206306 & 2683683553383 & 894561206306 & 894561140771 \end{bmatrix} \quad (10)$$

$v1$

$$\begin{bmatrix} -459459339518 \\ -894561206306 \end{bmatrix} \quad (11)$$

$F := \mathrm{abs}(v1[1])$

$$459459339518 \quad (12)$$

$G := \mathrm{abs}(v1[2])$

$$894561206306 \quad (13)$$

$rmin := \dfrac{(q + g \cdot F)}{G}$

$$1351417702001 \quad (14)$$

$\max(rmin, lb)$

$$1351417702001 \quad (15)$$

$is(\ Ub > 2 \cdot rmin)$

$$true \quad (16)$$

$r := lb + 250;$

$$1176477442250 \quad (17)$$

Encrypt with RCPKC−1

$e := h \cdot r + m\ \mathbf{mod}\ q$

$$1282633974950194452504 68 \quad (18)$$

Decrypt with RCPKC−1

$a := f \cdot e\ \mathbf{mod}\ q$

$$77119369025681764 \quad (19)$$

$m0 := a \cdot Fg\ \mathbf{mod}\ g$

$$14 \quad (20)$$

Figure S2. Screenshot of Maple code for RCPKC.1 encryption/decryption in settings of Example S3.

Example S4. *LBRA attack using GLR against RCPKC.1*

In this example, we try attacking RCPKC.1 using GLR Code S1. GLR terminates in 18 iterations finding $v1 = (F, G) = (-459459339518, -894561206306)$ that is neither (f, g) nor $(r, e-m)$ as shown in (21)-(25) Figure S3. Since gcd(F, q) =gcd$(F, G) = 2$, F has no inverses modulo q and G, and $v1$ cannot be used to decrypt the ciphertext. Thus, Let's try the second shortest vector $v2 = (-20749667184266511407213 3, 229534132287)$ that is neither (f, g) nor $(r, e-m)$ as shown in (26)-(30) Figure S3. When using (F, G) for decryption of e, we get, $m1 = 65549 \neq m = 14$ as shown in (31)-(33) Figure S3. Thus, actually, ciphertext decryption fails if using any of the shortest vectors returned by GLR.

It has been noticed by anonymous reviewer that the value of $F \cdot e = r \cdot G + F \cdot m$ could be negative, but still satisfy correctness decryption condition in the absolute value

$$|r \cdot G + F \cdot m| < q.$$

Therefore, we can see that

$$F \cdot e = r \cdot G + F \cdot m = 156494785800294925503676 = -1052431033814334249202500 \bmod q,$$

and $|-1052431033814334249202500| < q = 1208925819614629174706176$ as shown in (34) of Figure S3. Thus, decryption correctness condition (39) holds. On the other hand, $A = (F \cdot e \bmod q) - q = -1052431033814334249202500$. And the plaintext is restored as $m3 = (F^{-1} \bmod G) \cdot A \bmod G = -509 = 14 \bmod 523$, that is equal to $m = 14$ as shown in (35)-(37) Figure S3. Thus, the GLR attack succeeds revealing the plaintext message in the conditions of Example S3. Herein, it is necessary to be noticed that RCPKC.1 can be attacked by any of the short vectors returned by GLR. Maple code of this example is available in Example S4 of the supplementary material

Example S5. *Example of Finding RCPKC.2 Random Interval, and LBRA by GLR Failure*

This example aims to show the process of finding RCPKC.2 random interval, and how LBRA using GLR fails to compromise RCPKC.2 private key/message. For calculations, Maple is used. See Figure S4. Maple code of this example is available in Example S5 of the supplementary material

Key Creation

Let $mgLen = 16$, $qLen = 80$, meeting (38), $q = 2^{qLen}$, private key components, $g = 65,535$, and $f = 1,351,417,702,001$, are selected to meet (33) and (36) respectively as shown in (2) and (3) of Figure S4. Values F_q and F_g are found in (4) and (5) of Figure S4. The public key component, h, is calculated according to (10) as shown in (6) of Figure S4.

Finding Random Interval

To select random r, GLR algorithm shown in Code S1 is launched with inputs $V_1 = (1, h)$ and $V_2 = (0, q)$. GLR terminates in 18 iterations as shown in (8) of Figure S4, with 5 pairs (F_i, G_i) satisfying (44) shown in (7) of Figure S4, it is noticed that none of these vectors is equal to (f, g). Hence, (45) is satisfied. Maximum F_i and minimum G_i are found in (9), and (10) of Figure S4; value $rmin$ is defined according to (46) as shown in (11), $rmin$ also satisfies (47) as shown in (18) of Figure S4. $rmax$ is calculated in (12) of Figure S4. After calculating $max(\alpha \cdot 2^{qlen/2}, rmin)$ in (13) of Figure S4, it is perceived that (51) is satisfied as shown in (14) of Figure S4. Thus, r is selected from (50) as shown in (15) of Figure S4.

LBRA using GLR Failure

For the message $m = 14$, it is noticed that decryption correctness condition (49) is valid using private key (f, g) as shown in (16) of Figure S4, and not valid for (F_i, G_i) returned by GLR as shown in (17) of Figure S4. Hence, GLR attack fails to return keys usable for ciphertext decryption.

$GLR(f, g, h, q);$

$$\begin{bmatrix} 1133958117 & 459459339518 & 26960316553 & 459459339518 & 891958362483 \\ 44728059201205 & 894561206306 & 2683683553383 & 894561206306 & 894561140771 \end{bmatrix} \quad \textbf{(21)}$$

$counter$

$$18 \quad \textbf{(22)}$$

$v1$

$$\begin{bmatrix} -459459339518 \\ -894561206306 \end{bmatrix} \quad \textbf{(23)}$$

$F := v1[1]$

$$-459459339518 \quad \textbf{(24)}$$

$G := v1[2]$

$$-894561206306 \quad \textbf{(25)}$$

$gcd(F, q);$
$gcd(F, G);$

$$2$$
$$2 \quad \textbf{(26)}$$

$v2$

$$\begin{bmatrix} 891958362483 \\ -894561140771 \end{bmatrix} \quad \textbf{(27)}$$

$F := v2[1]$

$$891958362483 \quad \textbf{(28)}$$

$G := v2[2]$

$$-894561140771 \quad \textbf{(29)}$$

$gcd(F, G \cdot q)$

$$1 \quad \textbf{(30)}$$

$igcdex(F, q, 'Finvq', 'qz') :$
$igcdex(F, G, 'FinvG', 'Gz') :$
$Finvq;$
$FinvG;$

$$-2074966718426651140721 33$$
$$229534132287 \quad \textbf{(31)}$$

$A := F \cdot e \ \mathbf{mod} \ q$

$$1564948167966083188061 88 \quad \textbf{(32)}$$

$m1 := FinvG \cdot A \ \mathbf{mod} \ G$

$$65549 \quad \textbf{(33)}$$

$is(|r \cdot G + F \cdot m| < q)$

$$true \quad \textbf{(34)}$$

$A := F \cdot e \ \mathbf{mod} \ q$

$$156494816796608318806188 \quad \textbf{(35)}$$

$A := A - q$

$$-105243100281802085589998 8 \quad \textbf{(36)}$$

$m2 := A \cdot FinvG \ \mathbf{mod} \ G$

$$14 \quad \textbf{(37)}$$

Figure S3. GLR attack against RCPKC.1 in settings of Example S3

```
mgLen := 16 :
qLen := 80 :
q := 2^qLen :
f := 1351417702001 :
g := 2^16 - 1 :
gcd(f, q·g)
                                1                                        (1)
is( g < 2^mgLen and g ≥ 2^(mgLen - 1))
                              true                                       (2)
alpha := 1.07 :
is(f < 2^(qLen - mgLen - 1) and f ≥ alpha·2^(qLen/2))
                              true                                       (3)
igcdex(f, q,'Fq','qz') :
igcdex(f, g,'Fg','gz') :
Fq
                      1542604047705809790798 25                          (4)
Fg
                              2291                                       (5)
h := Fq·g mod q;
                      417923022495305103287663                           (6)
GLR(f, g, h, q)
                                0
[      1133958117  459459339518   26960316553  459459339518  891958362483 ]
[  44728059201205  894561206306 2683683553383  894561206306  894561140771 ]   (7)
counter
                               18                                        (8)
maxF := max(result[1, 1..ColumnDimension(result)])
                          891958362483                                   (9)
minG := min(result[2, 1..ColumnDimension(result)])
                          894561140771                                   (10)
rmin := ceil((q + g·maxF)/minG)
                         1351417832690                                   (11)
rmax := floor(q/g - f)
                      18447024201563593104                               (12)
max(trunc(alpha·2^(qLen/2)), rmin)
                         1351417832690                                   (13)
is(rmax > 2·max(trunc(alpha·2^(qLen/2)), rmin))
                              true                                       (14)
r := rmin + 1024;
                         1351417833714                                   (15)
m := 14 :
is(|r·g + f·m| < q)
                              true                                       (16)
F1 := result[1, 1] : F2 := result[1, 2] : F3 := result[1, 3] : F4 := result[1, 4] : F5 := result[1, 5] :
G1 := result[2, 1] : G2 := result[2, 2] : G3 := result[2, 3] : G4 := result[2, 4] : G5 := result[2, 5] :

is(|r·G1 + F1·m| < q or |r·G2 + F2·m| < q or |r·G3 + F3·m| < q or |r·G4 + F4·m| < q or |r·G5 + F5·m| < q)
                             false                                       (17)
is(h·rmin > q)
                              true                                       (18)
```

Figure S4. Screenshot of Maple code for Example S5.

2. Codes

Code S1. *Maple code of LBRA by GLR on CPKC private key/message*

Maple code of LBRA by GLR on CPKC private key/message returned as the shortest vector w=$v1$ of the lattice $E(V1, V2)$, where $V1$, $V2$ are from (24). This code is available in Code S1 in the supplementary material.

```
1.  GLR := proc (f, g, h, q)
2.  local fg, fgnorm, v1norm, v2norm, a, tmp, i;
3.  global result, counter, flag, v1, v2;
4.  flag := 0;
5.  v1 := Vector[column]([1, h]);
6.  v2 := Vector[column]([0, q]);
7.  fg := Vector[column]([f, g]);
8.  result := Matrix(2, 1);
9.  fgnorm := evalf(norm(fg, 2));
10. a := 1;
11. counter := 0;
12.  while a <> 0 do
13.   v1norm := evalf(norm(v1, 2));
14.   v2norm := evalf(norm(v2, 2));
15.   if v2norm < v1norm then
16.      tmp := v1;
17.      v1  := v2;
18.      v2 := tmp
19.   end if;
20.   if f = v1[1] or f = v2[1] then
21.      if g = v1[2] or g = v2[2] then
22.          flag := 1;
23.          print("keys are found")
24.      end if
25.   end if;
26.   if v1norm <= 10*fgnorm then
27.      result := <result| v1>;
28.   end if;
29.   if v2norm <= 10*fgnorm then
30.      result := <result| v2>
31.   end if;
32.   a := round(DotProduct(v1, v2)/norm(v1, 2)^2);
33.   v2 := v2-a*v1;
34.   counter := counter+1
35.   end do;
36. print(flag);
37. result := abs(result);
38. result := DeleteColumn(result, [1])
39. end proc
```

Note that lines $20-30$ in Code S1 are added to support the proposal of RCPKC in Section 5.
